# Amiodarone’s major metabolite, desethylamiodarone inhibits proliferation of B16-F10 melanoma cells and limits lung metastasis formation in an *in vivo* experimental model

**DOI:** 10.1371/journal.pone.0239088

**Published:** 2020-09-25

**Authors:** Zita Bognar, Anna Maria Cseh, Katalin Fekete, Csenge Antus, Rita Bognar, Antal Tapodi, Fadi H. J. Ramadan, Balazs Sumegi, Ferenc Gallyas

**Affiliations:** 1 Department of Biochemistry and Medical Chemistry, University of Pecs, Medical School, Pecs, Hungary; 2 MTA-PTE Nuclear-Mitochondrial Interactions Research Group, Pecs, Hungary; 3 Szentagothai Research Center, University of Pecs, Medical School, Pecs, Hungary; Duke University School of Medicine, UNITED STATES

## Abstract

Previously, we demonstrated the *in vitro* anti-tumor effects of desethylamiodarone (DEA) in bladder and cervix cancer cell lines. In the present study, we intended to establish its potentiality in B16-F10 metastatic melanoma cells *in vitro* and *in vivo*. We assessed cell proliferation, apoptosis and cell cycle by using sulforhodamine B assay, Muse™ Annexin V & Dead Cell and Muse® Cell Cycle assays, respectively. We determined colony formation after crystal violet staining. For studying mechanistic aspects, immunoblotting analysis was performed. We used a C57BL/6 experimental lung metastasis model for demonstrating *in vivo* anti-metastatic potential of DEA. DEA inhibited *in vitro* proliferation and colony formation, and *in vivo* lung metastasizing properties of B16-F10 cells. It arrested the cells in G0/G1 phase of their cycle likely via p21 in a p53-dependent fashion, and induced caspase mediated apoptosis likely via inversely regulating Bcl-2 and Bax levels, and reducing Akt and ERK1/2 activation. In this study, we provided *in vitro* and *in vivo* experimental evidences for DEA’s potentiality in the therapy of metastatic melanomas. Since DEA is the major metabolite of amiodarone, a worldwide used antiarrhythmic drug, safety concerns could be resolved more easily for it than for a novel pharmacological agent.

## Introduction

Desethylamiodarone (DEA), the major metabolite of the widely used antiarrhythmic drug amiodarone (AM) also has antiarrhythmic activity [1]. Both AM and DEA are strongly bound to plasma proteins [2]. During AM treatment, DEA rapidly accumulates in extracardiac tissues; sometimes in higher concentrations than AM itself does [3–5]. Tissue concentrations of AM and of DEA are hundred to more than thousand times higher than the corresponding plasma concentrations of 1.6–5.3 μM for AM, and 1.7–4.5 μM for AM plus DEA [6]. The most affected are adipose, liver and lung tissues, but skin, pancreas, myocardium and thyroid gland also massively accumulate these drugs. Except for the adipose tissue, concentration of the metabolite are usually higher than that of the parent molecule following chronic administration of AM [7]. Repetitive exposures of cell cultures to AM and DEA resulted in a cumulative and partially saturable uptake. Under all conditions tested, DEA accumulation was higher than that of AM [8]. The mean elimination half-life was found to be about 40 days and varies considerably between individuals [9].

The therapeutic concentration of AM has been recommended to be < 5.7 μM [10]. However, antiarrhythmic AM therapy is limited by the toxic side effects of both the parent molecule and DEA [11]. These side effects manifest in cardial, ocular, pulmonary, hepatic, dermatologic, hematological, psychiatric, thyroid and neuromuscular adverse reactions, and chronic AM treatment even can cause epididymitis and syndrome of inappropriate antidiuretic hormone secretion [11]. Based on its tissue accumulation properties and toxic effects, we proposed its potentiality in cancer therapy [12, 13]. In this study, we investigated DEA’s therapeutic potentiality in metastatic melanoma since new effective and safe treatments for this type of cancer are urgently needed.

Clarifying melanocyte biology, relevant oncogenic mutations as well as involved molecular signaling pathways on melanomagenesis have expanded our knowledge about melanoma remarkably in the past three decades [14]. However, melanoma is still the most lethal form of skin cancer accounting for approximately 132,000 new cases each year [15]. Despite the inflation of therapeutically approaches, metastatic melanoma still has a very poor prognosis, with a five-year survival rate of 15.1% [15, 16]. The prognosis even worse when the tumor has disseminated to distant sites and visceral organs [17]; the median survival time is only 6–9 months and the 3-year survival rate is about 10–15% [18]. Additionally, metastatic melanoma is highly resistant to chemotherapy. Most conventional chemotherapy agents have failed because of the patients’ low response rates [19] and significant toxicity of the agents [20, 21] emphasizing the importance of finding novel therapeutic tools. Accordingly, in this study we investigated the effect of DEA on growth, apoptosis of B16-F10 melanoma cells, and on lung metastasis formation in an *in vivo* experimental model.

## Materials and methods

### Materials

Protease inhibitor cocktail and all chemicals for cell culture were purchased from Sigma–Aldrich Co. (Budapest, Hungary). DEA was a gift from Professor Andras Varro (Department of Pharmacology and Pharmacotherapy, University of Szeged, Szeged, Hungary). The following primary antibodies were used: anti-Bcl-2, anti-Bax, anti-caspase 3 (clone H-277), anti-poly(ADP-ribose) polymerase 1 (PARP-1), anti-Akt, anti-phospho-Akt (Ser^473^), anti-extracellular signal-regulated kinase (ERK1/2) (Thr^202^/Tyr^204^), anti-phospho-ERK1/2 (Thr^202^/Tyr^204^), anti-p53, anti-p21, anti-p27, anti-cyclin dependent kinase (CDK)2, anti-cyclin D1 each 1:500 dilution and anti-glyceraldehyde-3-phosphate dehydrogenase (GAPDH) (1:2000, clone 6C5). Antibodies were purchased from Cell Signaling Technology (Beverly, MA, USA) except caspase 3, PARP-1, which were bought from Santa Cruz Biotechnology (Wembley, UK), while anti-GAPDH antibody was obtained from EMD Millipore Bioscience (Darmstadt, Germany).

### Cell culture

Metastatic melanotic B16-F10 mouse melanoma cell line was purchased from the American Type Culture Collection (LGC Standards, Wesel, Germany). Cells were cultured in Dulbecco’s modified Eagle’s medium supplemented with 10% fetal bovine serum and an antibiotic solution (1% penicillin and streptomycin mixture) (Life Technologies, Darmstadt, Germany). Cells were maintained in a humidified environment at 37°C with 5% CO_2_. They were split twice weekly for up to a maximum of 10 weeks. Cells were tested by MycoAlert^TM^ Plus Mycoplasma detection kit monthly.

### Cell proliferation assay

B16-F10 cells (10^5^/well) were cultured in 96-well cell culture plates overnight and treated with different concentrations (2.5–10 μM) of DEA. The attached cells were fixed in situ with addition of cold 10% trichloroacetic acid solution at 0, 24, 48 and 72 hours after treatment. Cell number (absorbance) was then estimated by the sulforhodamine B (SRB) assay, as previously described [22]. Proliferation was assessed by comparing light absorbance measured at the first time and was presented as fold values. The measurements were performed in quadruplicates and repeated three times.

### Cell viability assay

B16-F10 cells (3 × 10^5^/well) were plated in 24-well plates, cultured overnight and treated with the indicated concentration of DEA for 24, 48 or 72 hours. Cell viability after DEA treatment was quantified using the Muse™ Cell Count & Viability Assay, and the flow cytometry-based Muse™ Cell Analyzer (EMD Millipore Bioscience) according to the instructions provided by the manufacturer. Cell viability was expressed as the relative percentage of living cells of the control samples. The experiments were performed in quadruplicate and repeated three times.

### Colony formation assay

B16-F10 cells were plated in triplicates into 6-well plates at a starting density of 500 cells/well, before treating them with different concentrations of DEA. After 7 days of incubation, the cells were washed and stained with crystal violet and the colonies with diameter of > 0.5 mm were counted. The number of colonies was determined and normalized to the number of colonies in the controls. All experiments were repeated three times.

### Detection of apoptosis

For quantitative analysis of apoptosis, we used the Muse™ Annexin V & Dead Cell Assay on a Muse™ Cell Analyzer. The assay utilizes annexin V to detect phosphatidylserine on the external membrane of apoptotic cells. B16-F10 cells at a starting density of 1 × 10^6^ cells were seeded onto regular plates and treated for 24 hours without or with the indicated concentrations of DEA. The cells were harvested, and 100 μL of cell suspension was added to 100 μL of the Muse™ Annexin V & Dead Cell reagent. After 20 minutes of incubation at room temperature in the dark, the samples were analyzed according to the manufacturer’s protocol. All experiments were repeated three times.

### Cell cycle assay

After treatment with DEA (10 μM) for 24 hours, B16-F10 cells were collected by centrifugation at 300 x g for 5 minutes, washed with ice-cold phosphate buffered saline (PBS), fixed with 70% ethanol, stained with a premixed reagent composed of the nuclear DNA intercalating stain propidium iodide (PI) and RNAse A in a proprietary formulation, and analyzed according to the manufacturer’s protocol. PI discriminates cells at different stages of the cell cycle, based on differential DNA content in the presence of RNAse to increase the specificity of DNA staining. The Muse Cell Cycle Software Module performs calculations automatically. All experiments were repeated three times.

### Immunoblot analysis

B16-F10 cells at a starting density of 1 × 10^6^ cells were seeded into regular plates and treated as for the cell viability assay. Cells were harvested at intervals in a chilled lysis buffer containing 0.5 mM sodium-metavanadate, 1 mM EDTA and protease and phosphatase inhibitor cocktails (1:200), all purchased from Sigma–Aldrich Co. Cell lysates were boiled and subjected to 10% sodium dodecyl sulfate polyacrylamide gel electrophoresis before being transferred to nitrocellulose membranes. The membranes were blocked in 5% low-fat milk for 1.5 hours at room temperature before being exposed to primary antibodies at 4°C overnight in a blocking solution. Appropriate horseradish peroxidase-conjugated secondary antibodies were used at a dilution of 1:5000 (anti-mouse and anti-rabbit IgGs; Sigma–Aldrich Co.) and visualized by enhanced chemiluminescence (Amersham Biosciences, Piscataway, New Jersey, USA). The films were scanned, and the pixel volumes of the bands were determined using NIH Image J software (Bethesda, Maryland, USA). For membrane stripping and reprobing, the membranes were washed in a stripping buffer (0.1 M glycine, 5 M MgCl_2_, pH 2.8) for an hour at room temperature. After washing and blocking, the membranes were incubated with primary antibodies for nonphosphorylated or loading control proteins. All experiments were repeated three times.

### Ethics

Animal experiments were conducted in strict accordance with the recommendations in the Guide for the Care and Use of Laboratory Animals of the National Institutes of Health. The experimental protocol was approved by the Animal Research Review Committee of the University of Pécs, Hungary (Permit number: BA02/2000-5/2017).

### Mouse pulmonary metastasis model

Six weeks old male C57BL/6 mice were bred and maintained at the Department of Biochemistry and Medical Chemistry, University of Pecs, Medical School. All animals were housed 3 or 4 per cage, under controlled laboratory conditions (22 ± 1°C, 50 − 60% relative humidity and 12/12 hour light-dark cycles) with free access to water and standard rodent chow. Paper tunnels were used for environmental enrichment.

We used the pulmonary metastasis model that is widely used as an *in vivo* test for assessing antitumor efficacy of medications [23]. B16-F10 cells (5 x 10^5^/0.1 ml) were injected into the lateral tail vein of mice using 30G 1/2 needle and 1-ml syringe. There were no changes observed in motility or food intake in tumor bearing animals during the experiment. All animals were checked at least once daily for aspects of general health including activity, posture and fur grooming, body condition score was also assessed as previously described [24].

The mode of DEA administration and dosage were determined based on the study by DeWitt et al. [25]. Prior to the study, safety of DEA treatment was confirmed by administration of 25 mg/kg DEA to 5 mice for two weeks. Mice were randomly divided into 2 groups of 6 mice each. Each mouse was given a daily intraperitoneal injection of either 100 μl 0.9% saline solution containing 10% ethanol as the vehicle control or 25 mg/kg DEA. Treatment was started one day after cell injection and was given every third day, lasted for consecutive 16 days after injection when the animals were weighed and sacrificed by cervical dislocation under isoflurane (AbbVie Ltd., Budapest, Hungary) anesthesia. The lungs was removed, rinsed in PBS, and weighed. The lung mass index was calculated as the ratio of lung weight to body weight. The harvested lungs were fixed in 4% formalin. Tumor nodules on the surfaces of the lungs were counted under a stereomicroscope. Then, the whole lung was embedded in optimum cutting temperature compound (Sakura Finetek, USA), sectioned (12 μm thickness), and stained with hematoxylin and eosin. Histological observations were performed under a microscope (BX51, Olympus, Japan) by an expert blind to the experiment. Percentage of the total area occupied by tumor was measured using the Panoramic viewer 1.15.4 (3DHISTECH, Hungary).

### Data analysis

All data are expressed as mean ± standard deviation (SD). The concentration-dependent effects of DEA in each experiment were tested with ANOVA using the post hoc Dunnett test. We used the Mann-Whitney U test to compare the treated example to the vehicle-treated control. Differences were considered significant at values of p ≤ 0.05. Statistical analyses were performed using IBM SPSS Statistics v20.0 (IBM Corporation, New York, USA).

## Result

### Effects of DEA on cell viability

**Fig 1:**
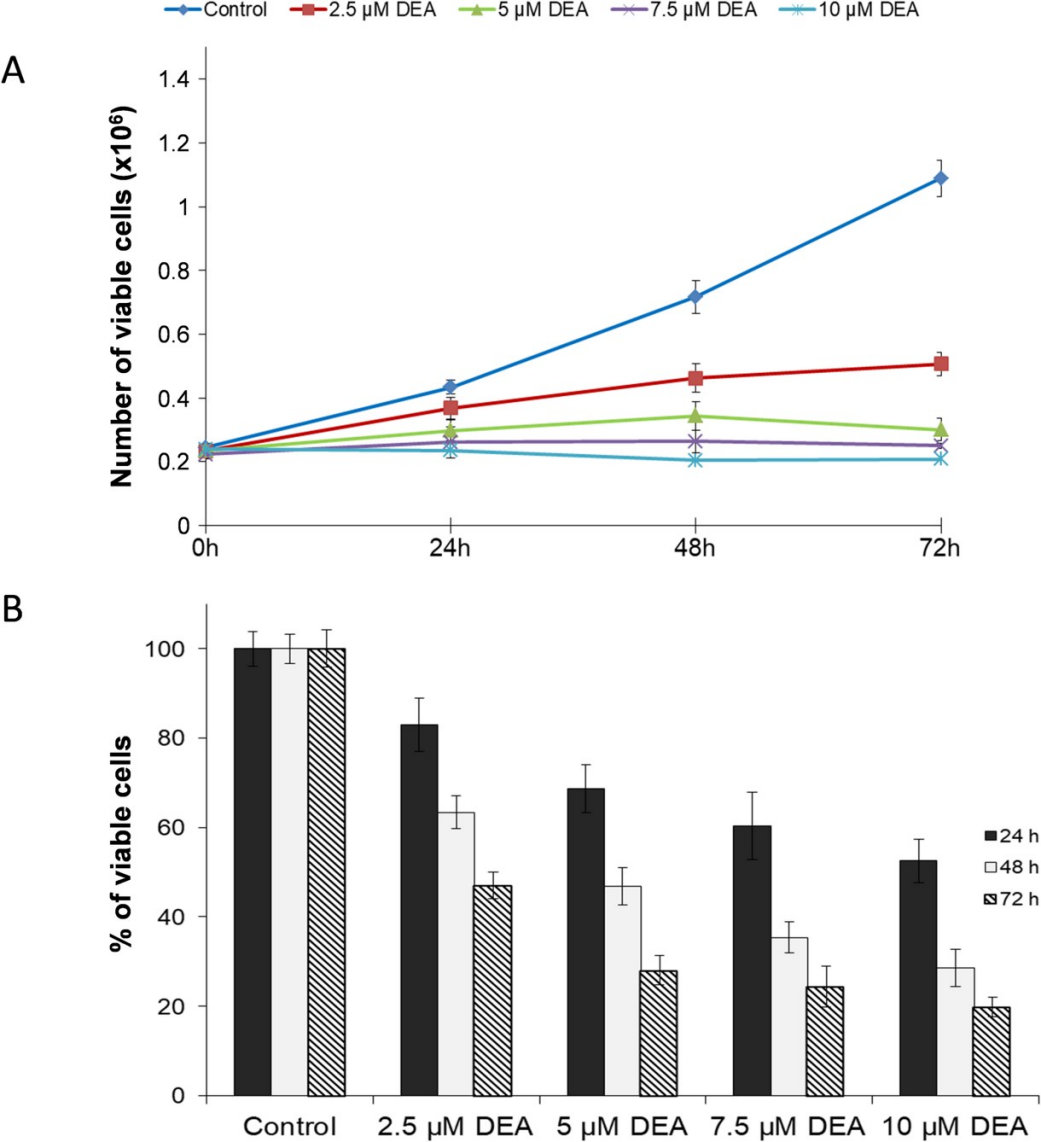
Effect of DEA on cell proliferation and viability of B16-F10 murine melanoma cells. The cells were incubated with the indicated concentrations of DEA for 24, 48 and 72 hours. Controls were treated with vehicle (0.2% DMSO). Cell proliferation and viability were assessed by using the SRB assay (A) and the Muse™ Cell Count & Viability Assay (B). Data represent means ± SD of three independent experiments performed in at least quadruplicate. Data were analyzed using ANOVA with Dunett post hoc test. ** p ≤ 0.01 and *** p ≤ 0.001 compared to the corresponding control group.

B16-F10 murine melanoma cells in monolayer culture were treated with increasing concentrations of DEA for 24, 48 and 72 hours. DEA had a statistically significant antiproliferative and cell death-inducing effect on the cells determined by using the SRB assay (Fig 1A) and the Muse™ Cell Count & Viability Assay (Fig 1B). The vehicle-treated cells served as negative controls. These data indicate that DEA induces cell death in a dose- and time-dependent manner; therefore, the possible pathways contributing to DEA-induced cell death were further analyzed.

### Effect of DEA on colony formation

**Fig 2:**
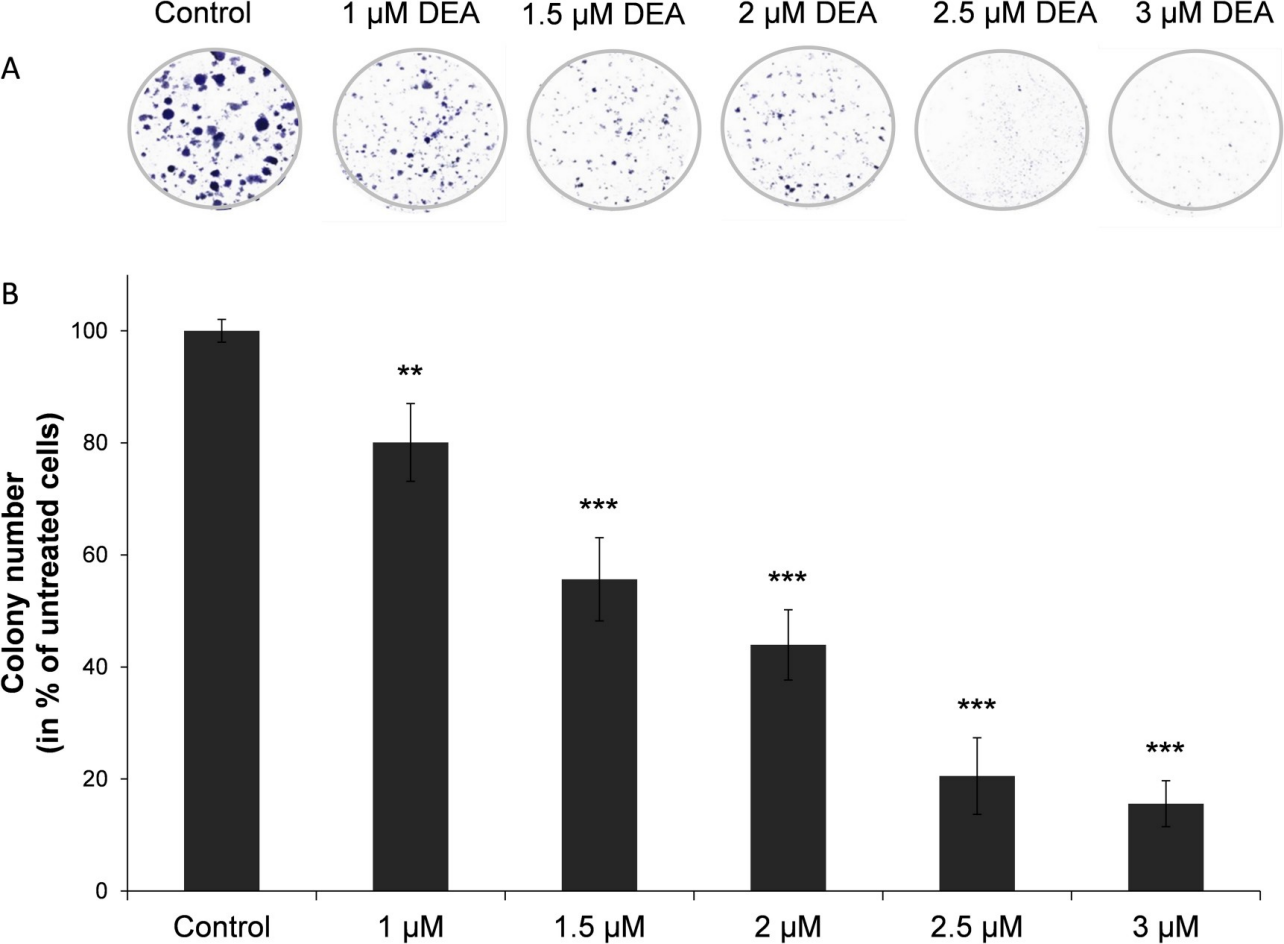
Effect of DEA on colony formation of B16-F10 murine melanoma cells. For the assay the cells were exposed to increasing concentrations of DEA for 7 days. The results are presented as representative images (A) and as a bar diagram (B). Controls were treated with vehicle (0.2% DMSO). The results are mean ± SD of three independent experiments performed in at least quadruplicate. Data were analyzed using ANOVA with Dunett post hoc test. ** p ≤ 0.01 and *** p ≤ 0.001 compared to the control group.

To assess DEA’s effect on colony forming ability of the B16-F10 cells, a long-term assay was performed. We treated the cells with 0–3 μM DEA for 7 days then colonies of > 0.5 mm were counted. As we found, DEA significantly decreased number and size of the colonies even at the lowest concentration used (Fig 2). These data indicate that DEA can induce cell death and can inhibit colony formation at low micromolar concentrations.

### Effect of DEA on activation of apoptosis in B16-F10 cells

**Fig 3:**
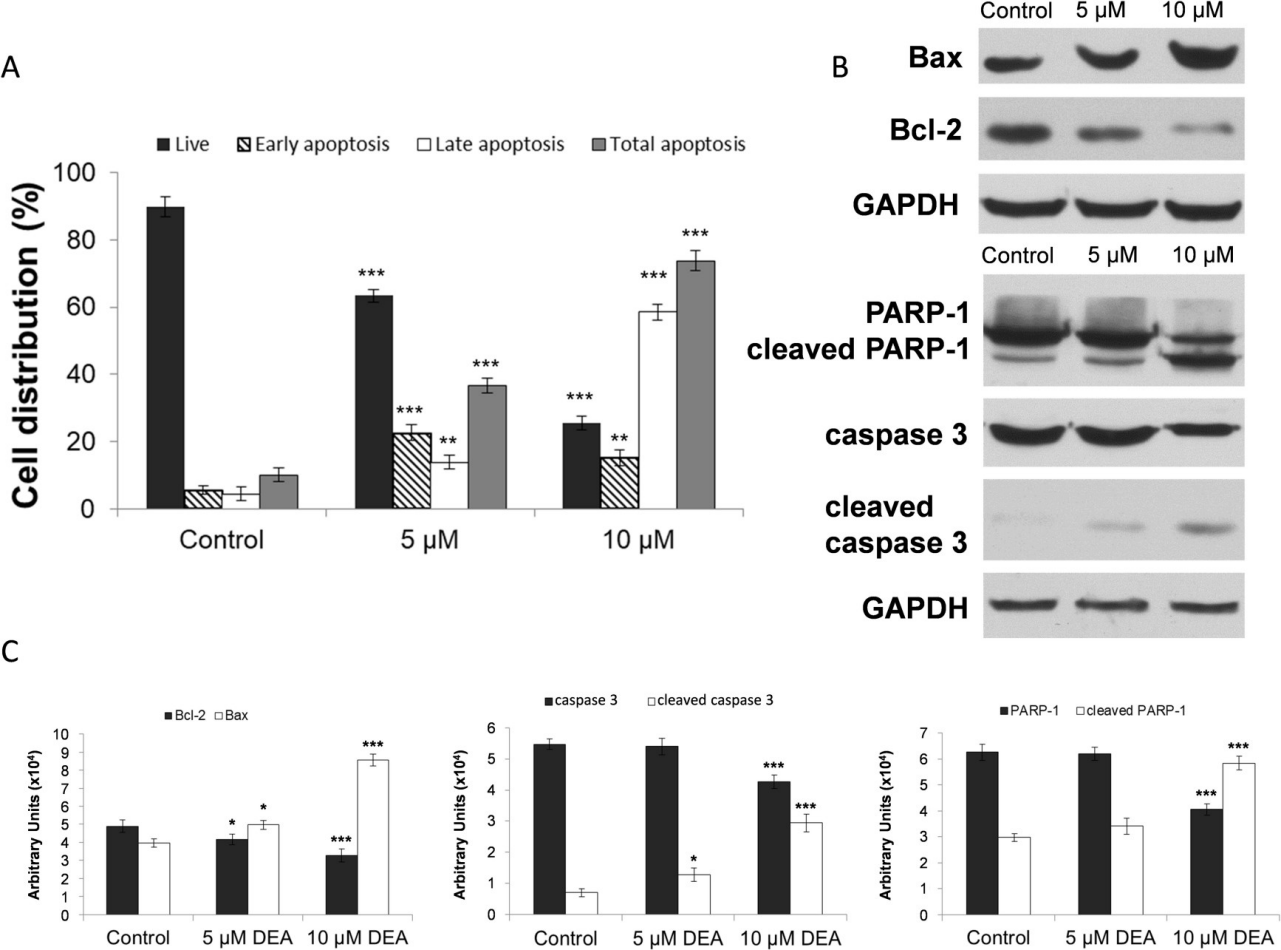
Effect of DEA on activation of apoptosis in B16-F10 cells. After subjected to 24 hours treatment with increasing concentrations of DEA the cells were stained with the Muse™ Annexin V & Dead Cell Reagent and measured on the Muse™ Cell Analyzer. (A) The bar chart shows the percent distribution of living (dark gray bars), early apoptotic (striped bars), late apoptotic (white bars) and total apoptotic cells (light gray bars). Controls were treated with vehicle (0.2% DMSO). (B) Immunoblot analysis on total cell lysates was performed using antibodies against Bax, Bcl-2, PARP-1, caspase 3 and cleaved caspase 3. The results of the immunoblot analysis and the densitometric analysis of the immunoblots are presented in panels B and C. The experiments were repeated three times in at least quadruplicate, and the results are expressed as the mean ± SD. Data were analyzed using ANOVA with Dunett post hoc test (A) and with Mann-Whitney U test (C). * p ≤ 0.05, ** p ≤ 0.01 and *** p ≤ 0.001 compared to the corresponding control group.

Flow cytometry with the Muse™ Annexin V & Dead Cell Assay was used to investigate the mode of DEA induced cell death. The assay utilizes cell surface annexin V binding that measures appearance of phosphatidylserine on the plasma membrane’s external surface, a marker of apoptosis. We observed that DEA increased the total apoptosis rate in a dose-dependent manner. We found a total apoptosis rate of 34.61 ± 2.17% for 5 and 73.71 ± 3.12% for 10 μM DEA in contrast to the control’s 10.11 ± 1.97% (Fig 3A). At the lower DEA concentration, rate of early apoptosis exceeded that of the late, however, at the higher concentration, late apoptosis predominated (Fig 3A).

### Effect of DEA on apoptosis related proteins in B16-F10 cells

To examine how treatment with DEA affects the steady-state levels of apoptosis related proteins, B16-F10 cells were treated with 5 and 10 μM of DEA for 24 hours, then the cells were lysed, and immunoblot analyses were performed on the extracted proteins. We found that the expression of Bcl-2 was significantly decreased as compared with control while the expression of Bax was significantly increased (Fig 3B and 3C). Additionally, DEA dose-dependently activated the effector caspase, caspase 3, which activation was confirmed by corresponding cleavage of PARP-1, a target of caspase 3 (Fig 3B and 3C). These data indicate that DEA predominantly activates apoptotic cell death via several pathways that can be an advantage in cancer therapy, where apoptotic pathways can be inactivated during the development of cytostatic resistance.

### Effect of DEA on the cell cycle in B16-F10 cells

**Fig 4:**
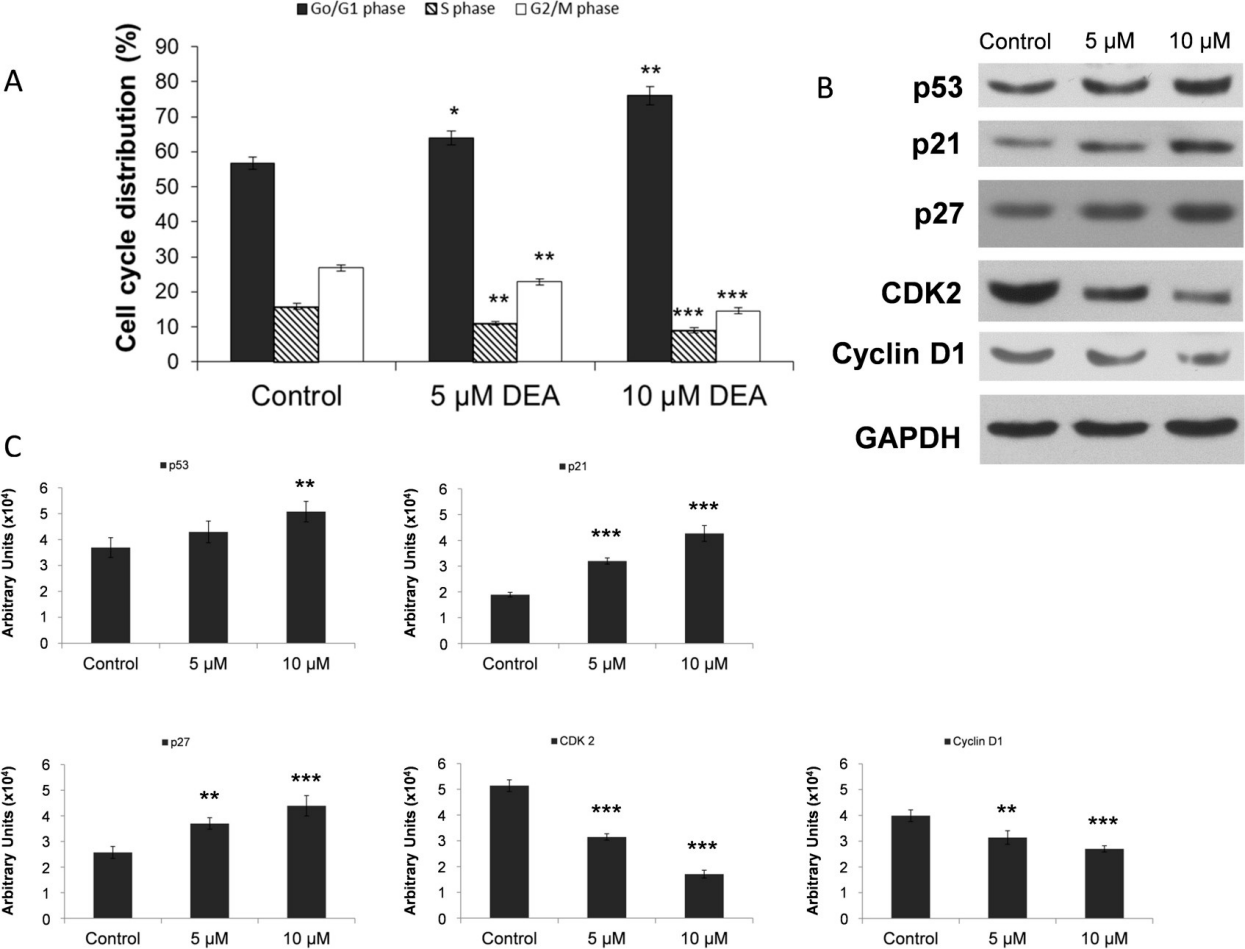
Effect of DEA on the cell cycle in B16-F10 melanoma cells. The cells were treated with different concentrations of DEA (0, 5 and 10 μM) for 24 hours. Controls were treated with vehicle (0.2% DMSO). Then they were harvested, fixed with ethanol and stained with propidium iodide. DNA content was determined by Muse™ Cell Analyzer. The results are presented as a bar diagram, percentage of cells in G0/G1 (dark grey bars), S (striped bars) and G2/M phases (white bars) of the cell cycle (A). Proteins from parallel cells were extracted for immunoblot analysis performed by using antibodies specific to p53, p21, p27, CDK2 and cylcin D1. GAPDH was used as a loading control. The results are presented as representative immunoblots (B) and densitometric analysis of immunoblots in bar diagrams (C). The results are mean ± SD of three independent experiments performed in at least quadruplicate. Data were analyzed using ANOVA with Dunett post hoc test (A) and with Mann-Whitney U test (C). * p ≤ 0.05, ** p ≤ 0.01 and *** p ≤ 0.001 compared to the corresponding control group.

Cell proliferation is the net result of cell division and cell death. Although the data presented in Fig 3 suggest that DEA limits proliferation of B16-F10 by inducing predominantly apoptotic cell death, we performed cell cycle analysis to study the other aforementioned aspect of proliferation. We treated B16-F10 cells with 5 and 10 μM DEA for 24 hours, and found that the percentage of cells in G0/G1 phase significantly increased from 56.75 ± 1.73% (0 μM) stepwise to 63.9 ± 1.94% (5 μM) and 75.91 ± 2.67% (10 μM). At the same time there was a decrease in the percentage of S and G2/M phase cells (Fig 4A). These data indicate that DEA at both concentrations induced cell cycle arrest in the G0/G1 phase that may contribute to its overall inhibitory effect on B16-F10 cell proliferation.

To investigate the mechanisms of DEA-induced cell cycle arrest, we examined the expression of cell cycle-related proteins by immunoblotting. DEA treatment (10 μM) for 24 hours, significantly increased p53, p21 and p27 while decreased CDK2 and cyclin D1 steady-state protein levels (Fig 4B and 4C).

### Effect of DEA on cytoprotective kinase signaling pathways

**Fig 5:**
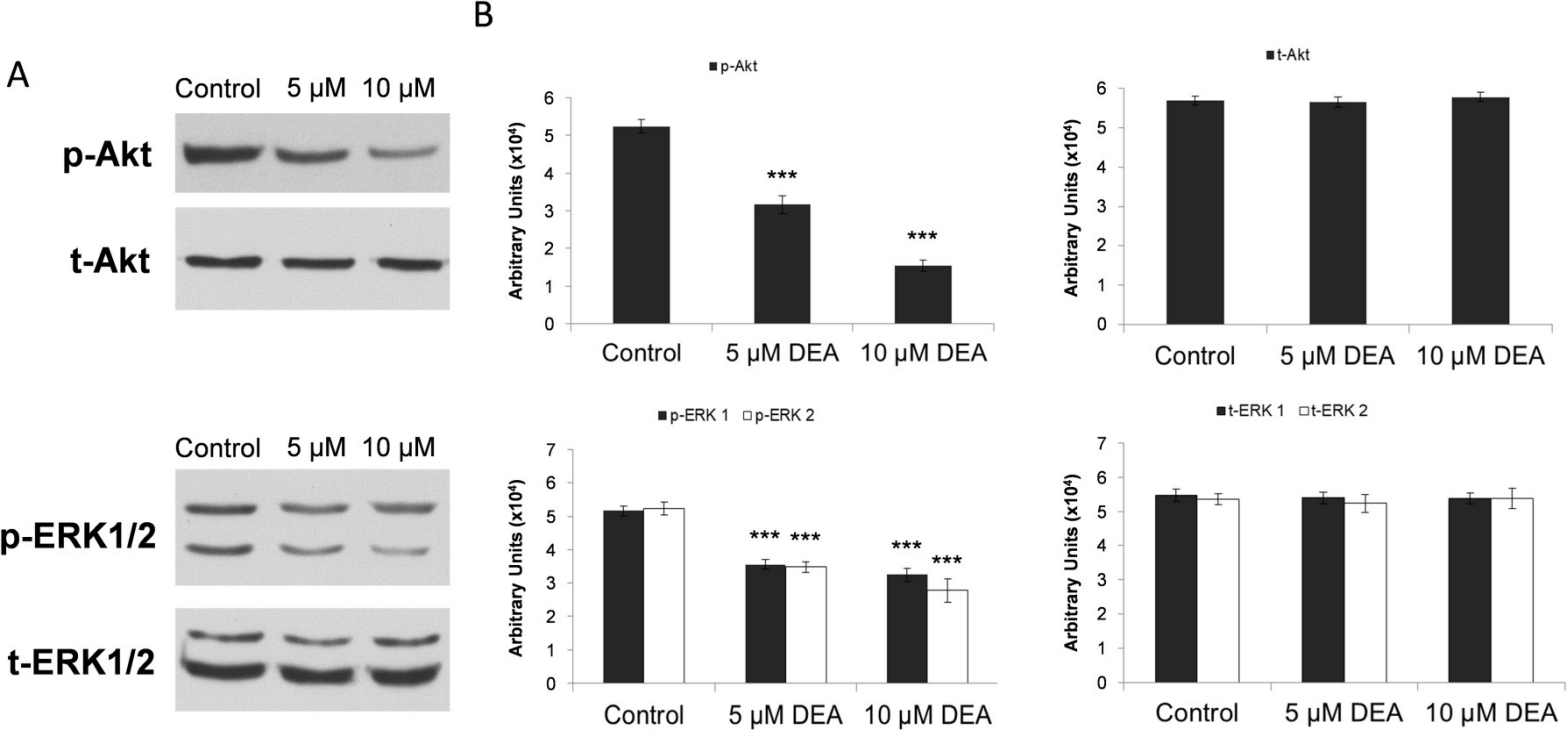
Effect of various DEA concentrations on the activation of cytoprotective signaling kinases in B16-F10 cells. Total cell extracts were analyzed by immunoblotting 24 hours after the DEA treatments. Activation of Akt and ERK1/2 as well as equal protein loading was confirmed by kinase specific primary antibodies: anti-Akt (t-Akt), anti-phospho-Akt (p-Akt), anti-ERK1/2 (t-ERK1/2) and, anti-phospho-ERK1/2 (p-ERK1/2). The figure shows the results of the immunoblots (A) and the densitometric analysis (B) of three independent experiments. Data are expressed as the mean ± SD, and presented as arbitrary unit. Data were analyzed using Mann-Whitney U test. *** p ≤ 0.001 compared to the control group.

ERK promotes cell proliferation, differentiation and survival, while Akt is considered as a major cell and tissue protecting pathway. Therefore, we wanted to know whether DEA affected activation of these kinases. To this end, we determined phosphorylation status of Akt, ERK1/2 in B16-F10 cells treated with 0–10 μM DEA by using phosphorylation specific primary antibodies and immunoblotting. As we found, DEA did not affect Akt or ERK1/2 steady-state protein level at either concentration used. However, there was a concentration-dependent down-regulation of Akt Ser^473^ phoshorylation upon DEA treatment (Fig 5A and 5B). Basically, identical results were found for ERK1/2 (Fig 5A and 5B).

### Effect of DEA on lung metastasis formation in an *in vivo* experimental model

**Fig 6:**
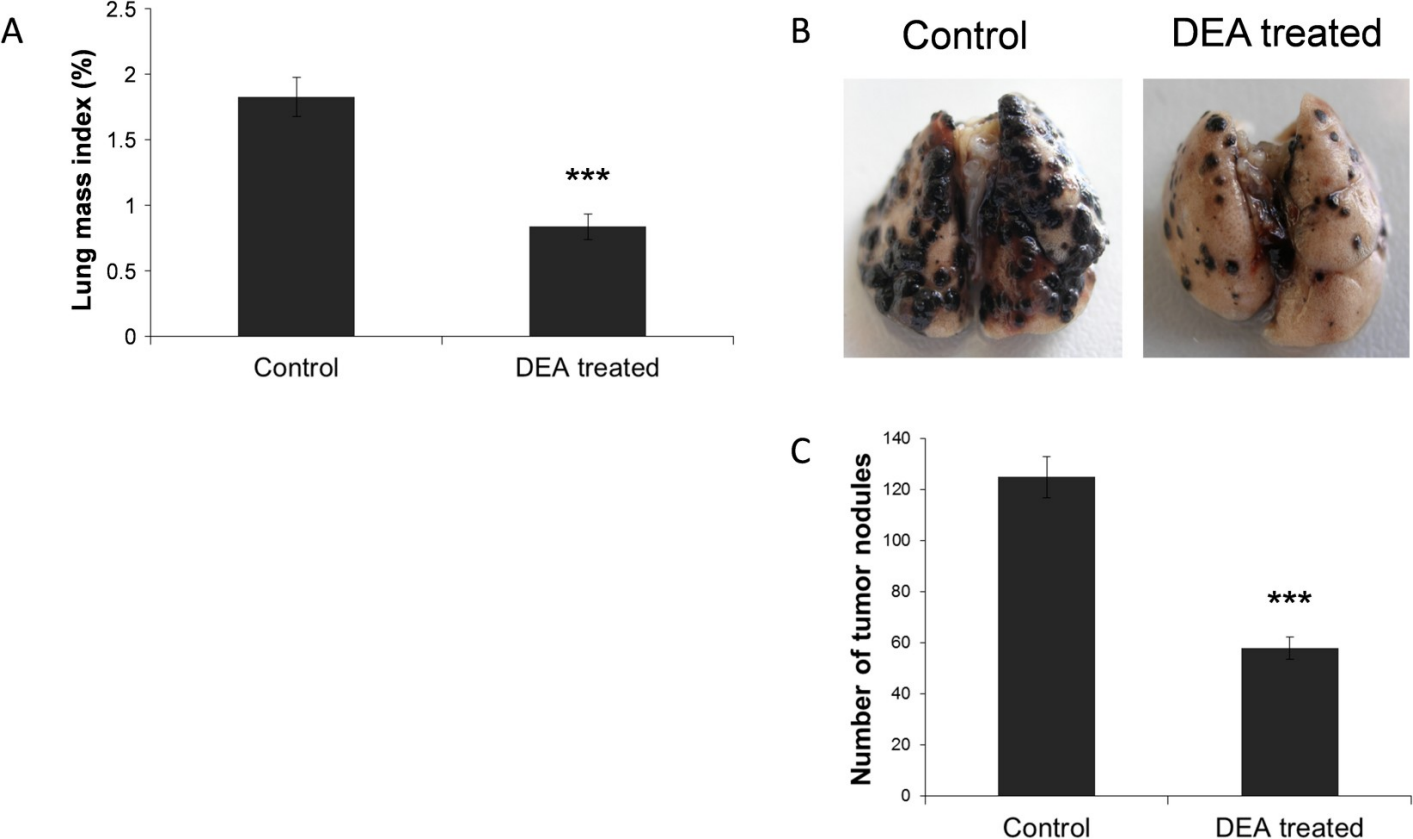
Effect of DEA on lung metastasis formation in vivo. Lung metastasis in C57BL/6 mice was induced by injecting B16-F10 cells into the lateral tail vein. Control animals received vehicle control (100 μl 0.9% saline solution containing 10% ethanol) 24 hours after the administration of the B16 F10 cells, while the DEA group was treated with 25 mg/kg DEA intraperitoneally (6 mice/group). Treatment was repeated every third day. At the 16^th^ day, the mice were sacrificed, the lungs were dissected, photographed and their mass was measured. Representative photos (A) of lungs of control and DEA treated animals are presented. Lung mass index (B) and the number of tumor nodules on the on lungs’ surface (C) are presented as bar diagrams, mean ± SD. Data were analyzed using Mann-Whitney U test. *** p ≤ 0.001 compared to control group.

To evaluate the effect of DEA on metastasis formation, we used an *in vivo* lung metastasis model. Murine melanoma B16-F10 cells were injected into the lateral tail vein of 6-weeks-old male C57BL/6 mice then they were divided into two groups (6 mice/group). Intraperitoneal administration of 25 mg/kg DEA or vehicle control was started 24 hours after tumor cell administration, and was repeated every third day. At the 16^th^ day of the experiment, the animals were sacrificed and the lungs were removed for analysis. The lungs were weighted and lung mass index was calculated (Fig 6A). After the lungs were fixed in 4% formalin and photographed (Fig 6B) and the number of tumor nodules on the lungs’ surface was also determined (Fig 6C). Accordingly, DEA treatment diminished lungs mass index and the number of tumor nodules (Fig 6A and 6C).

**Fig 7:**
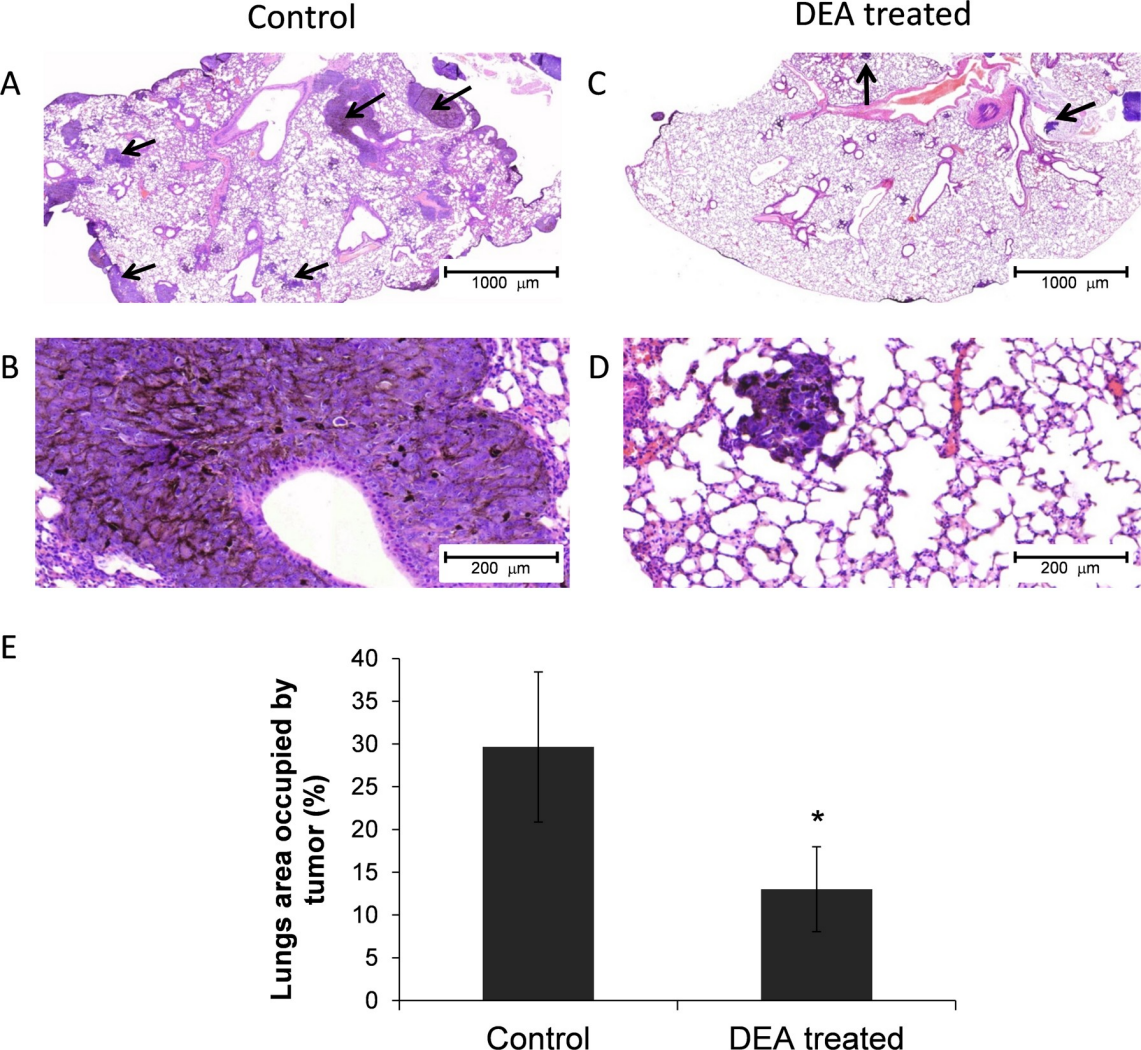
Histopathology analysis on lung tissue of mice injected with B16-F10 melanoma cells. Lungs of B16-F10 injected animals treated with vehicle control or 25mg/kg DEA every third day for 16 days were fixed in neutral buffered formalin, sectioned and stained with hematoxylin and eosin. Representative sections (A-D) demonstrate darkly stained tumor nodules (arrows). Metastasis formation was quantitatively assessed by determining the tumor area (E) as percentage of the total lung section area, means ± SD from 6 randomly selected section per lung by using the Pannoramic Viewer Imaging System. Data were analyzed using Mann-Whitney U test. * p ≤ 0.05 compared to control.

Additionally, histopathological analysis was performed by an expert who was blind to the experiment on lung tissue sections after hematoxylin and eosin staining. There were melanoma cells with poliedric morphology with a great amount of melanin content as cytoplasm granules or in a perinuclear distribution. Additionally, aberrant nodular proliferation in broncho-alveolar regions characteristic of epithelial melanoma was observed in all sections (Fig 7A–7D). However, there was a marked difference in tumor nodule pattern distribution and concentration between untreated and DEA treated animals. In the vehicle treated group, the nodules were of considerable size and were distributed in all part of the lung parenchyma (Fig 7A and 7B). In contrast, in the DEA group (Fig 7C and 7D), the tumor nodules were much smaller in size and were organized in a predominantly peripheral distribution (Fig 7C and 7D). To quantify the morphological observations, image analysis with the Pannoramic Viewer Imaging System was performed on randomly selected sections (6 per lungs), and tumor area as the percentage of lung section area was calculated. As we found, the tumor area was significantly decreased in the lung tissue of DEA *vs*. vehicle treated animals (Fig 7E). These results are in complete accordance with those of the macroscopic observations (Fig 6) and indicate that DEA can inhibit melanoma tumor metastasis *in vivo*.

## Discussion

Previously, we demonstrated that DEA significantly inhibited the proliferation and viability of T24 human transitional cell bladder carcinoma and HeLa human cervical cancer cell lines [12, 13]. It mitigated colony formation *in vitro*, as well as it induced apoptosis and G0/G1 phase cell cycle arrest in a dose-dependent manner [12, 13], exactly as it did in the present study in mouse B16-F10 melanoma cells (Figs 1–4). *In vitro* antiproliferative and apoptosis-promoting properties of a drug represent a rather poor translational value for its potentiality in human cancer therapy. On the other hand, a uniform effect on three very different, highly proliferative cell lines indicate that DEA interferes with fundamental survival promoting processes likely present in most cancer cell lines of different tissue and species origin.

A defective apoptosis signaling pathway has been considered a major cause of chemoresistance in melanoma. High expression of Bcl-2 is correlated with resistance to chemotherapy in human melanomas and other tumors [26]. Furthermore, in wild-type p53 expressing melanoma cell lines such as B16-F10, cytostatic agents induce G1 arrest and down-regulation of Bcl-2 [27–29]. In a complete agreement with this view, we found DEA to act as an apoptosis-stimulating factor for the melanoma cells presumably via upregulation of p53 and Bax, and downregulation of Bcl-2 (Figs 3 and 4). The resulting apoptosis likely involved mitochondrial mechanisms leading to caspase 3 activation as it was evidenced by cleavage of caspase 3 and its downstream target PARP-1 (Fig 3).

A number of evidence indicates that G1/G0 arrest occurs during the early steps of apoptosis in different cancer types following cytostatic treatment [30, 31]. For melanomas, regulators of the cell cycle’s G1 phase transition such as D and E type cyclins, CDK4/6, CDK inhibitors including p16 and p14, and retinoblastoma protein were suggested as diagnostic tools or therapeutic targets [13, 32–34]. Accordingly, we determined DEA’s effect on the cell cycle as well as on the metastasis inducer markers cyclin D1 and CDK2 [13, 35], the tumor suppressor p53 [36], and the cell cycle inhibitors p21 and p27 [37]. As we found, in a concentration dependent manner, DEA increased number of cells in the G0/G1 phase that was accompanied by reduced steady state levels of cyclin D1 and CDK2, and increased levels of p21, p27 and p53 (Fig 4). Because the p53 gene is not mutated in B16-F10 cells [27, 28], the observed induction of G1 phase arrest by DEA was likely mediated through p21, in a p53-dependent fashion.

Sustained activation of intracellular prosurvival signaling cascades such as the phosphatidylinositol 3-kinase/Akt pathway has been showed to significantly enhance cancer progression. Akt promotes cell survival and proliferation by suppressing apoptosis and stimulating cell cycle advancement [38, 39]. Progression and malignancy of various tumors is often associated with the constitutive activation of Akt [38, 40], which inactivates many proapoptotic proteins by phosphorylation [39]. The invasiveness of melanoma cells and their ability to form metastases may be related to the frequently observed high basal activity of Akt in these cells [41]. Our group showed that the high basal levels of active, phosphorylated Akt [38, 42] was decreased dose dependently by DEA treatment in B16-F10 cells (Fig 5), a highly invasive variant of B16 murine melanoma. This decrease in the activation of Akt may contribute to the effects of DEA on the cell cycle (Fig 4) and apoptosis (Fig 3).

The ERK pathway participates in the phosphorylation over 250 cellular substrates [43] thereby regulating various cellular processes such as cell adhesion, cell cycle progression, migration, survival, differentiation, metabolism, proliferation, and transcription [44]. Its activation was reported to induce cell cycle entry and G1 progression by upregulating proliferative transcription factors [45] and downregulating antiproliferative genes [46]. Accordingly, in melanomas, apoptotic resistance and metastatic property was reported to be associated with ERK pathway activation [47, 48]. Similarly to Akt, we observed a high basal level of activated ERK1/2 in the B16-F10 melanoma cell line that was diminished by DEA treatment in a dose dependent manner again without affecting total ERK1/2 steady-state level (Fig 5). Like in the case of Akt, attenuation of ERK1/2 activation could likely contribute to DEA’s effect on cell cycle (Fig 4) and apoptosis (Fig 3).

Metastasis is fundamental property of malignant cancer cells by which a certain cancer spreads from the location at which a tumor first arises to distant locations in the body [49, 50]. Cancer recurrence by metastasis is responsible for about 90% of mortality in cancer patients [51] and is currently a main target for cancer therapy. Metastasizing proceeds in a series of sequential steps including invasion, intravasation, survival and translocation in the circulation system, extravasation and survival in a new organ [52]. Within its limitations, the B16-F10 lung metastasis model [53] mimics all but the first two steps of this process. During the period of 16 days, the B16-F10 injected into tail vein of the animals formed a number of large tumor loci in the lungs (Fig 6) that occupied about 30% of tissue area in the lungs sections (Fig 7). Both number and size of the tumor loci were reduced by DEA (Figs 6 and 7) indicating that the drug has potentiality in reducing *in vivo* metastasis formation. However, the B16-F10 cells are of mouse tissue culture and not of human primary tumor origin. Additionally, they have been selected for their metastasizing property, and therefore have low immunogenicity [54]. Accordingly, further studies are needed preferably by using primary human melanomas to establish DEA’s potentiality in the therapy of metastatic melanomas.

## Conclusion

In this study, we provided *in vitro* and *in vivo* experimental evidences for DEA’s potentiality in the therapy of metastatic melanomas. Since DEA is the major metabolite of amiodarone, a worldwide used antiarrhythmic drug, safety concerns could be resolved more easily for it than for a novel pharmacological agent.

## Supporting information

S1 File (ZIP)
