# Supplementary figures and images for: Amiodarone’s major metabolite, desethylamiodarone inhibits proliferation of B16-F10 melanoma cells and limits lung metastasis formation in an in vivo experimental model
Source: PLoS One. 2020 Sep 25;15(9):e0239088. doi: 10.1371/journal.pone.0239088 (PMC7518930; doi:10.1371/journal.pone.0239088)

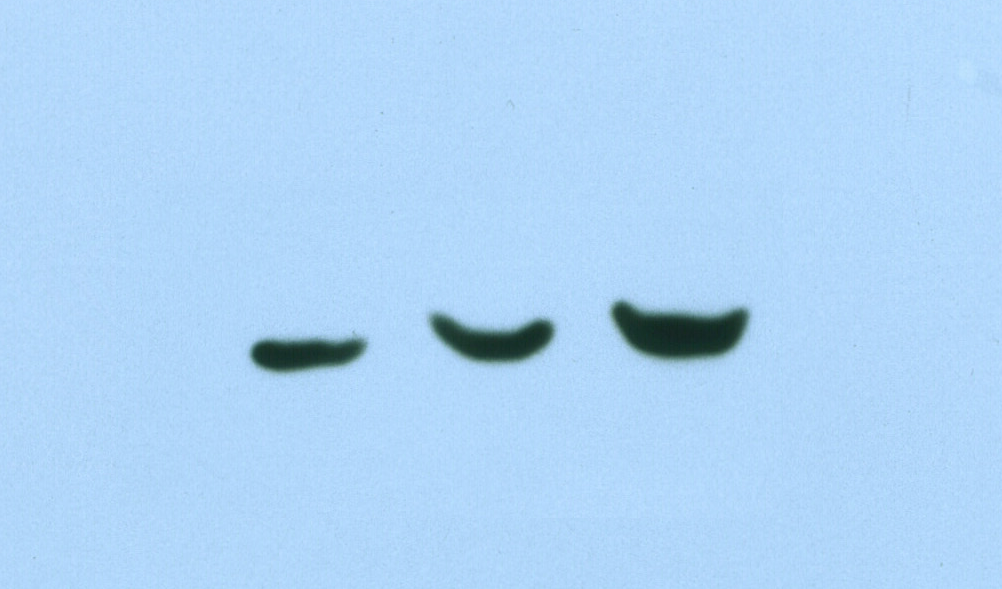

Supplement: S1 File — (ZIP) [file pone.0239088.s001.zip › Bax.tif]

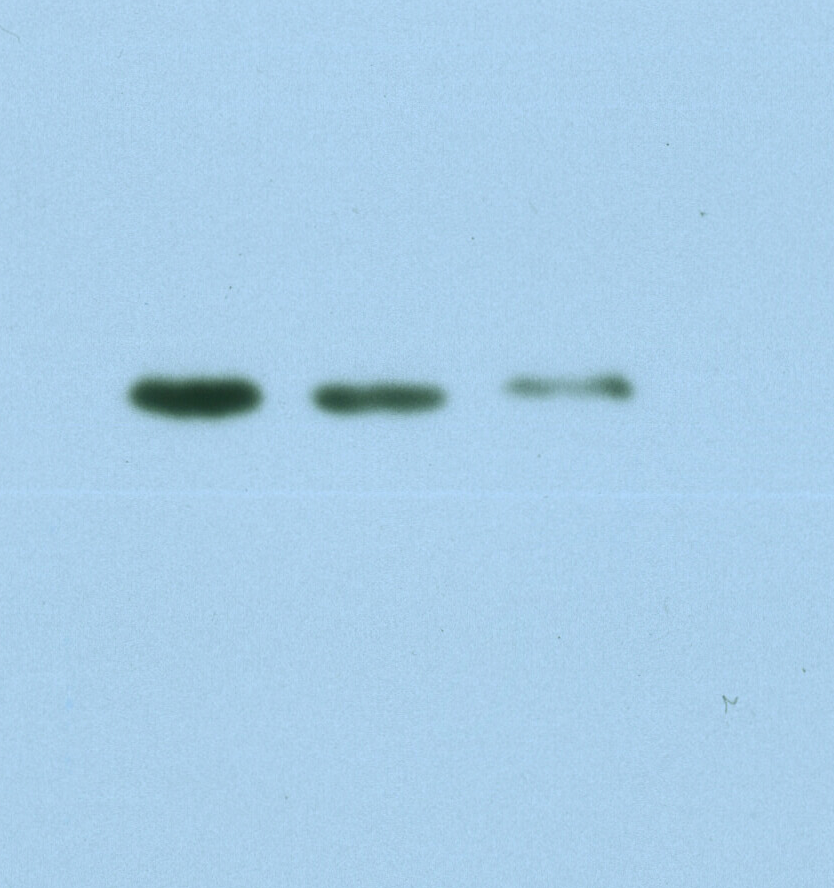

Supplement: S1 File — (ZIP) [file pone.0239088.s001.zip › Bcl-2.tif]

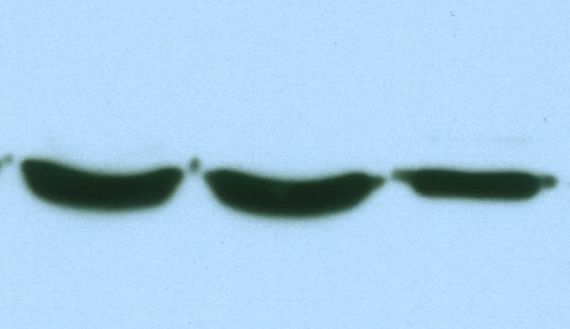

Supplement: S1 File — (ZIP) [file pone.0239088.s001.zip › Casp3.tif]

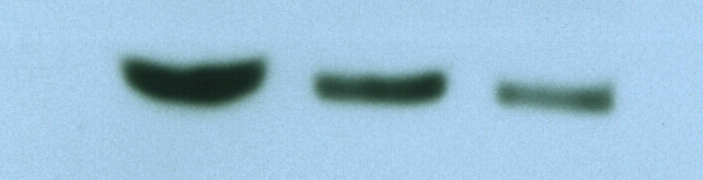

Supplement: S1 File — (ZIP) [file pone.0239088.s001.zip › CDK2.tif]

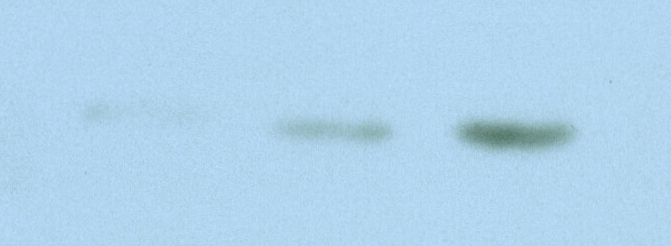

Supplement: S1 File — (ZIP) [file pone.0239088.s001.zip › cleaved caspase.tif]

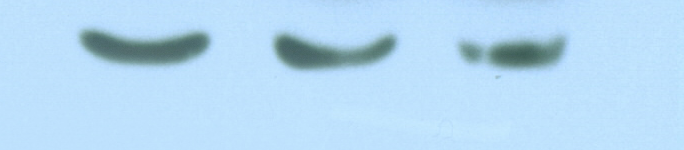

Supplement: S1 File — (ZIP) [file pone.0239088.s001.zip › Cyclin D1.tif]

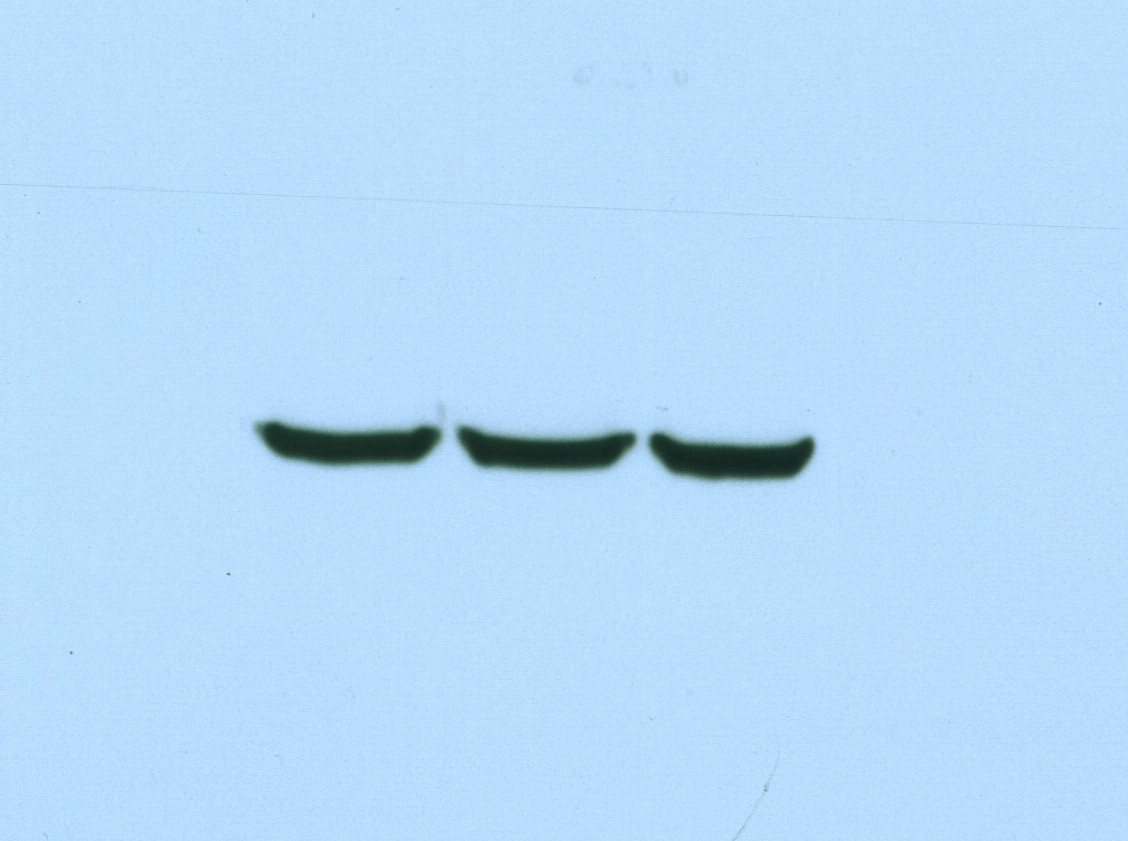

Supplement: S1 File — (ZIP) [file pone.0239088.s001.zip › GAPDH Fig3.tif]

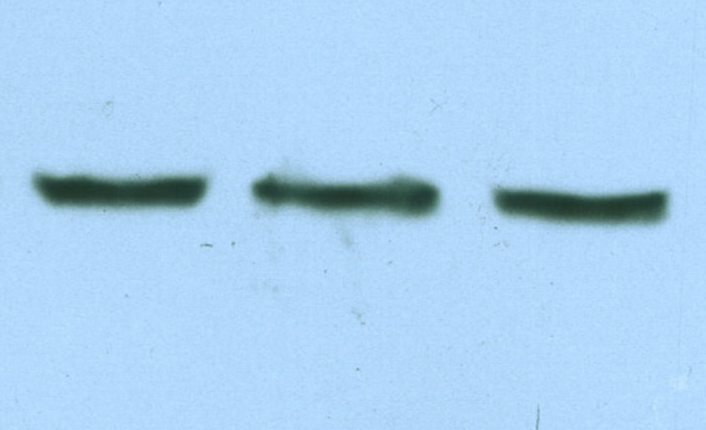

Supplement: S1 File — (ZIP) [file pone.0239088.s001.zip › GAPDH Fig3 2.tif]

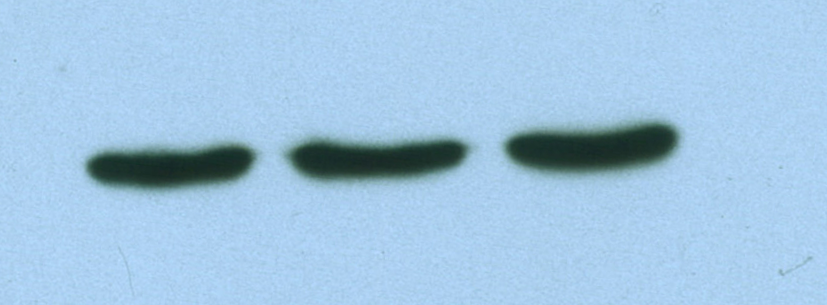

Supplement: S1 File — (ZIP) [file pone.0239088.s001.zip › GAPDH Fig4.tif]

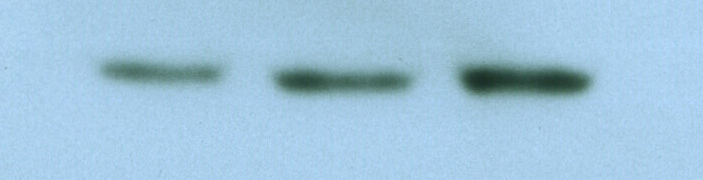

Supplement: S1 File — (ZIP) [file pone.0239088.s001.zip › p21.tif]

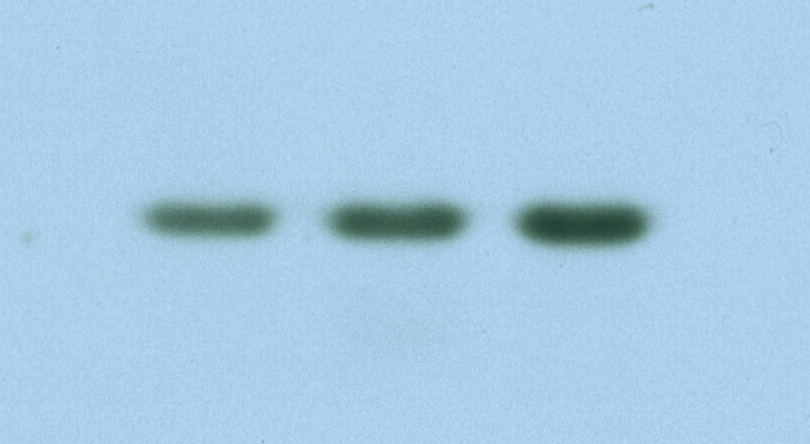

Supplement: S1 File — (ZIP) [file pone.0239088.s001.zip › p27.tif]

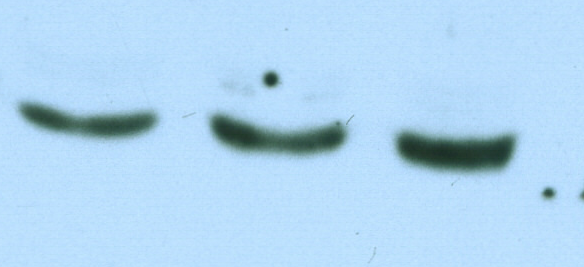

Supplement: S1 File — (ZIP) [file pone.0239088.s001.zip › p53.tif]

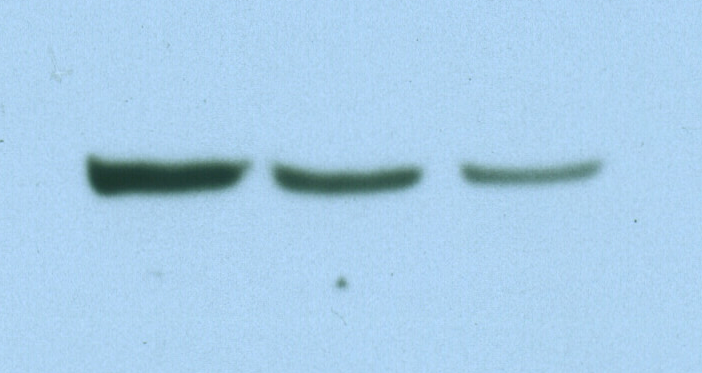

Supplement: S1 File — (ZIP) [file pone.0239088.s001.zip › pAkt.tif]

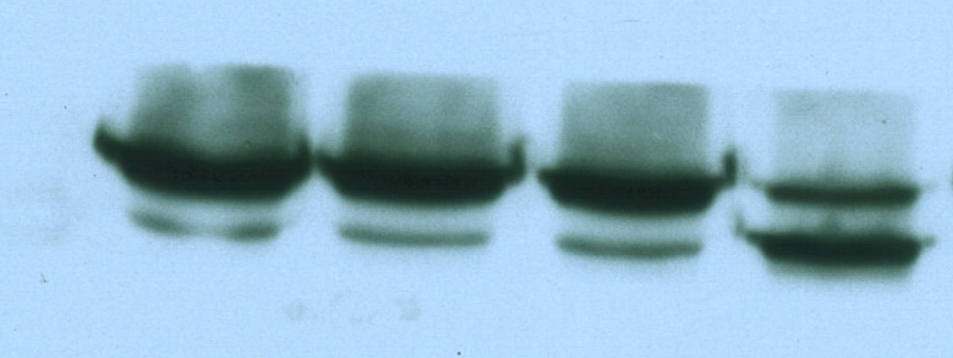

Supplement: S1 File — (ZIP) [file pone.0239088.s001.zip › PARP.tif]

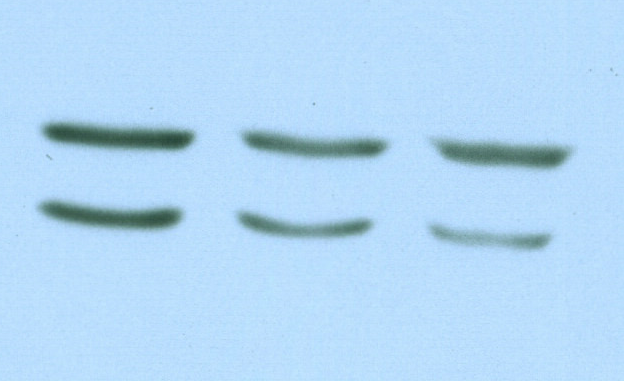

Supplement: S1 File — (ZIP) [file pone.0239088.s001.zip › pErk.tif]

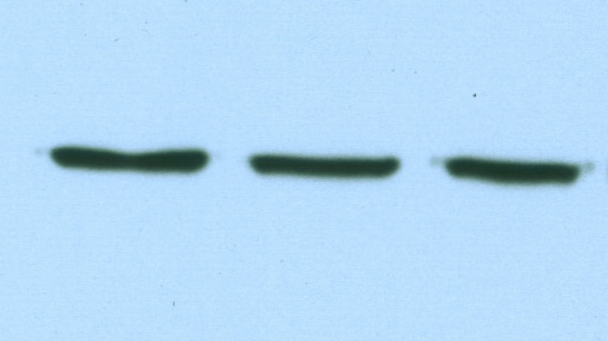

Supplement: S1 File — (ZIP) [file pone.0239088.s001.zip › tAkt.tif]

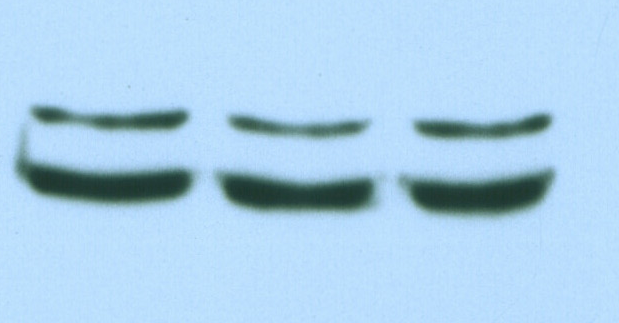

Supplement: S1 File — (ZIP) [file pone.0239088.s001.zip › tErk.tif]
